# Beyond the bone marrow: a review of therapeutic approaches for extramedullary disease in multiple myeloma and the significance of MRD assessment

**DOI:** 10.25122/jml-2025-0104

**Published:** 2025-06

**Authors:** Daniela Diaconescu, Dan Sebastian Soare, Cristina Elena Marinescu, Georgiana Elena Ene, Horia Bumbea

**Affiliations:** 1 Bone Marrow Transplantation Unit, Emergency University Hospital, Bucharest, Romania; 2 Scientific Research Methodology and Hematology, Carol Davila University of Medicine and Pharmacy, Bucharest, Romania

**Keywords:** plasmacytoma, extramedullary disease, multiple myeloma, minimal residual disease, novel therapies

## Abstract

Extramedullary disease (EMD) in multiple myeloma (MM) represents a distinct clinical entity associated with poor prognosis, therapeutic resistance, and aggressive behavior. EMD can occur at diagnosis or during relapses, either contiguous with bone lesions or as soft tissue plasmacytomas due to hematogenous spread. This review outlines the current understanding of EMD pathophysiology, diagnostic challenges, and therapeutic approaches. The review differentiates between bone-related and non-bone-related EMD, highlighting their prognostic implications. Diagnostic strategies rely on advanced imaging modalities, including PET-CT and MRI, and require histopathological confirmation through biopsy and immunohistochemistry. Management includes local therapies, primarily radiotherapy and, in selected cases, surgery, alongside systemic treatments involving proteasome inhibitors, immunomodulatory drugs, and monoclonal antibodies. New emerging therapies, such as chimeric antigen receptor T cells (CAR-T) and bispecific antibodies, are under evaluation for the treatment of relapsed/refractory EMD. Autologous stem cell transplantation is recommended for eligible patients, with tandem procedures considered in high-risk cases. The role of minimal residual disease (MRD) monitoring is emphasized, employing next-generation sequencing (NGS), flow cytometry, and imaging, with MRD negativity serving as a surrogate marker for treatment efficacy and survival prediction. Despite therapeutic advances, the prognosis for patients with EMD remains unfavorable. The review underscores the necessity of a multidisciplinary approach for accurate diagnosis, individualized treatment, and consistent monitoring. Recognizing EMD as a high-risk MM variant mandates the integration of novel diagnostics and therapies. Future clinical trials must incorporate EMD-specific endpoints to optimize treatment and improve outcomes.

## INTRODUCTION

Multiple myeloma (MM) is one of the most frequent malignant diseases, accounting for approximately 1% of all newly diagnosed cancers in Europe and about 15% of blood cancers, making it the second most prevalent hematologic malignancy [1]. The disease is characterized by clonal proliferation of plasma cells within the bone marrow, leading to end-organ damage defined by the CRAB criteria: hypercalcemia, renal insufficiency, anemia, and bone lesions. In addition to bone marrow involvement, MM may present with or evolve into extramedullary disease (EMD), characterized by the growth of clonal plasma cells outside the bone marrow microenvironment. The presence of EMD in MM is indicative of a biologically aggressive phenotype and is consistently associated with an unfavorable prognosis [2]. EMD can be identified either at initial diagnosis or during disease, particularly at relapse. Its occurrence is frequently correlated with high-risk cytogenetic abnormalities and elevated serum lactate dehydrogenase (LDH) levels [3]. These factors contribute to a more refractory disease course, characterized by a higher likelihood of multiple relapses and reduced responsiveness to therapy. Despite the availability of novel therapeutic agents, EMD remains a major clinical challenge, necessitating tailored treatment strategies and closer monitoring [4].

The management of extramedullary disease in multiple myeloma requires a personalized approach, with treatment regimens being adapted based on the anatomical site, extent of dissemination, and underlying disease biology. Management of extramedullary disease varies depending on its location and extent. In localized forms, radiotherapy alone may be sufficient, whereas in other cases, a combined approach involving radiotherapy and surgical excision is required [5]. When extramedullary involvement occurs in the context of systemic multiple myeloma, treatment typically includes systemic therapy with various combinations of anti-myeloma agents. Current guidelines recommend autologous stem cell transplant (ASCT) or tandem ASCT in selected cases [6]. Imaging techniques play a critical and irreplaceable role in the detection of extramedullary disease and in monitoring treatment response. Positron emission tomography-computed tomography (PET-CT), whole-body low-dose computed tomography (WBLD-CT), and magnetic resonance imaging (MRI) are the cornerstone imaging modalities in the evaluation of extramedullary disease, providing synergistic data on both the anatomical distribution and functional activity of myeloma lesions.

This review aims to provide a comprehensive analysis of EMD in multiple myeloma, encompassing its definition, classification, diagnosis, therapeutic strategies, clinical implications, and monitoring strategies in this field.

## EXTRAMEDULLARY DISEASE IN MULTIPLE MYELOMA – DEFINITION, CLASSIFICATION

The extramedullary disease is defined by the presence of monoclonal plasma cell proliferation occurring outside the bone marrow microenvironment, frequently manifesting as a localized tumor mass resulting from the accumulation of malignant cells in soft tissues [7]. Also referred to as extramedullary plasmacytoma, this manifestation can occur in a wide range of soft tissue sites, including the head and neck region, as well as the respiratory and gastrointestinal tracts. It is the result of hematogenous dissemination of clonal plasma cells and is strictly confined to soft tissues, with no involvement of adjacent bone structures [8].

Extramedullary plasmacytomas are thought to originate from plasma cells residing in mucosal-associated lymphoid tissue. Plasmacytomas are classified into two distinct entities: bone plasmacytoma and extramedullary plasmacytoma [9], each with unique clinical behavior and prognostic implications. Bone plasmacytomas arise within the medullary cavity and may present either as solitary bone plasmacytomas, typically involving the axial skeleton or other bones, or as multiple bone lesions, which are often considered manifestations of systemic disease [10,11].

Understanding the definition of extramedullary disease in multiple myeloma is critical, as it directly influences the selection of optimal, personalized therapeutic strategies—both in routine clinical management and within research settings [12].

To distinguish between the various types of plasmacytomas, the International Myeloma Working Group (IMWG) has established the following criteria [13]:

- **Solitary plasmacytoma of bone (SPB):** a single bone lesion composed of clonal plasma cells, in the absence of serum or urine M-protein, without bone marrow involvement characteristic of MM, no additional lesions on skeletal imaging, and no evidence of end-organ damage.
- **Extramedullary plasmacytoma (EMP):** similar to SPB, but involving an extramedullary (soft tissue) tumor consisting of clonal plasma cells rather than a bone lesion.
- **Multiple solitary plasmacytomas (MSP):** The presence of two or more bone or extramedullary lesions composed of clonal plasma cells without detectable M-protein in serum or urine, a normal bone marrow examination, a negative skeletal survey (aside from the lesions), and no end-organ damage.

An overview of extramedullary multiple myeloma is presented in Table 1. The high histological grade and the presence of angiogenesis in plasmacytoma increase the risk for progression to multiple myeloma [14,15]. Extramedullary plasmacytoma is sometimes considered an intermediate stage between monoclonal gammopathy of undetermined significance (MGUS) and overt MM [16] or a distinct aggressive subtype of MM. This aggressive behavior has been linked to specific cytogenetic abnormalities, including deletion 17p [17,18], TP53 deletions [19], and translocation t(4;14**)** [20], particularly when EMD presents alongside MM. The risk of progression from EMP to MM is estimated to range between 10% and 30% [21]. Furthermore, patients exhibiting bone lesions in combination with marked hypercalcemia are considered at elevated risk for developing extramedullary disease [2]. Among the molecular mediators, interleukin-6 (IL-6) has emerged as a key growth factor in plasma cell dyscrasias [14]. Elevated IL-6 levels are frequently observed in cases of extramedullary involvement, where IL-6 is thought to promote plasmacytoma development and proliferation of malignant plasma cells [14,22].

**Table 1:** Classification and definition of extramedullary multiple myeloma

Extramedullary multiple myeloma	Definition	Clinical presentation	Incidence
**Bone related plasmacytoma**	Plasmacytomas formed within the bone, contiguous with its marrow	Masses affecting axial skeleton: skull, stern, ribs, vertebrae, pelvic bones	<5% of patients with plasma cell disorders [14]
**Plasma cell leukemia**	Aggressive multiple myeloma – >20% or > 2 x 10^9^/L circulating plasma cells	Blood involvement. Frequently associated with extramedullary disease	2-3% of all plasma cell cancers [23]
**Extramedullary disease**	Infiltration of plasma cells or soft tissue plasmacytoma distant from the bone marrow (hematogenous spread)	Affected areas like lymph nodes, CNS, liver, pleura	14.5% of cases at time of diagnosis and 76% of patients at time of relapse [24]
**Solitary plasmacytoma**	Collection of abnormal plasma cells in bone or soft tissue without systemic disease	No involvement in skeleton or bone marrow, no CRAB criteria	3% of patients [25]

CNS, Central Nervous System; CRAB, Hypercalcemia, Renal Failure, Anemia, Bone Lesions; SP, Solitary Plasmacytoma [25].

## INCIDENCE

The incidence of MM has increased over recent years [26], accompanied by a parallel rise in the rates of extramedullary plasmacytoma and solitary plasmacytoma, particularly among the elderly population [9,27]. The underlying causes of EMP development remain incompletely understood; however, several factors, including genetic predisposition, viral infections, and radiation exposure, have been proposed as potential contributors [28].

The incidence of extramedullary disease in MM varies depending on the timing of diagnosis and disease stage. At initial diagnosis, so-called primary EMD has been reported in up to 20% of MM cases [29]. Other studies suggest lower rates at presentation, ranging from 6% to 10%, with a notable increase to 13% to 26% as the disease progresses or relapses [30]. This upward trend, particularly in relapsed or refractory cases, highlights the possibility of distinct mechanisms of clonal evolution or treatment resistance that may contribute to extramedullary spread [30].

## CLASSIFICATION OF EMD

In multiple myeloma, extramedullary disease is classified into two types (Table 1), depending on its relationship with the bone structure. The first type, bone-related EMD, involves plasmacytomas contiguous with bone, typically arising from bone lesions that extend into adjacent soft tissue. The second type, soft tissue-related EMD, results from hematogenous dissemination, leading to the formation of plasmacytomas in soft tissues without direct contact with bone.

The prognostic implications of these subtypes remain a subject of ongoing debate. Some studies suggest that soft tissue plasmacytomas may be associated with better overall survival compared to bone-related lesions [31]. However, other research indicates the opposite, showing that non-bone-associated EMD is linked to a worse prognosis than bone-related EMD [10].

### Diagnosis and staging evaluation of extramedullary disease

#### Challenges in diagnosis

The diagnosis of extramedullary disease in multiple myeloma can be very difficult, and there may be delays due to varying locations or subtle presentations [32]. An overview of the diagnostic criteria for plasmacytomas is presented in Table 2. Extramedullary plasmacytomas can be detected in all types of soft tissues, resulting in nonspecific symptoms that can delay diagnosis, ultimately leading to delays in specific treatment [33]. The symptoms that arise may be attributed to other conditions, which can increase the risk that patients delay seeking evaluation by a hematologist. Frequently, initial consultations occur with internal medicine specialists, which can further prolong the time to a definitive diagnosis of multiple myeloma [34].

**Table 2:** Diagnostic criteria of solitary bone plasmacytoma and solitary extramedullary plasmacytoma [40]

Solitary bone plasmacytoma	Solitary extramedullary plasmacytoma
One single area of bone destruction	Single extramedullary mass with clonal plasma cells
Normal histologically bone marrow aspirate/trephine	Histologically normal marrow aspirate or trephine
No other lesions on skeletal survey	No other lesions on skeletal survey
No CRAB criteria	No CRAB criteria
Low/absent serum or urinary level of monoclonal immunoglobulin	Absent/low serum or urinary level of monoclonal immunoglobulin
No lesions on the MRI scan of the spine	

In approximately 80–90% of cases, extramedullary plasmacytomas involve the head and neck regions, as well as the respiratory and gastrointestinal systems [35]. The patients can present symptoms such as headache, dysphagia, sore throat, epistaxis, or nasal obstruction. The involvement of other uncommon regions like the larynx can cause dysphonia, dysphagia, wheezing, and airway obstruction [28]. When the gastrointestinal tract is affected, patients may present with epigastric pain, anorexia, abdominal pain, or gastrointestinal bleeding. Mesenteric involvement is extremely rare, with fewer than ten cases described in the literature [36-38]. Similarly, pulmonary plasmacytomas may mimic lung neoplasms, presenting with nodular opacities (parenchymal or perihilar), mediastinal lymphadenopathy, or even alveolar damage [39].

#### Laboratory findings

When assessing diagnostic criteria, M protein should not be detectable in immunofixation of the serum or urine. Although these criteria are well known, approximately 50% of patients still have a detectable small M-protein in their urine or serum [13,41]. All other blood tests, such as urine/serum protein electrophoresis, peripheral blood cell count, blood calcium, renal function, and uninvolved immunoglobulins, are typically within normal ranges and should be performed in every case to rule out multiple myeloma. Patients with extramedullary plasmacytoma have no or under 5% bone marrow involvement. Extramedullary disease, on the other hand, is associated with laboratory tests that show an aggressive state of the disease: elevated lactate dehydrogenase (LDH) levels, high-risk cytogenetics abnormalities (deletions, translocations), presence of M-protein, particularly free light chains in urine, and bone marrow infiltration [42]. These findings collectively highlight the aggressive nature and poor prognosis associated with EMD. Additionally, mutational testing often reveals *RAS/BRAF* mutations, which are likely critical in the development of EMD [43].

#### Histology

A biopsy is crucial for understanding cellular characteristics in the diagnosis of extramedullary plasmacytomas while also excluding other malignancies. Performing a biopsy is necessary to identify plasma cell infiltration and characterize the phenotype of the cells. Histopathological analysis confirms the presence of plasma cell infiltration, while immunohistochemistry (IHC) provides critical insight into cellular phenotype and clonality. Key IHC markers used include CD138, CD38, and MUM1, which help establish the plasma cell origin of the tumor [44]. In association with biopsy, the exclusion of MM must be done to ensure that extramedullary plasmacytoma is not in the context of a systemic disease. Bone marrow aspiration or trephine biopsy might be necessary to correlate findings.

#### Imaging techniques

Imaging techniques play a crucial role in diagnosis and monitoring the extramedullary disease in multiple myeloma. Table 3 outlines the three principal imaging modalities currently used in the evaluation of MM/EMD patients [35,45].

**Table 3:** Imaging techniques in multiple myeloma [53]

	WBLD-CT	FDG PET/CT	Whole-body DWI-MRI
Accessibility	Fast-scanning, cheap, widely available, total body evaluation	60 minutes scanning time, more expensive, less available/reimbursed whole body technique, needs specialist for interpretation	Scanning time 30-60 minutes, more expensive, relatively reimbursed or available, axial and whole-body technique, requires specific expert to interpret
Radiation exposure	Low radiation dose (3-4 mSv), no need for IV contrast administration	Higher (6-10 mSv)	No radiation
Bone lesion evaluation	Lytic lesions > 5mm	Detects present lytic bone lesions or EMD and disease metabolism	Early bone damage
Favorite target	Used for CT-guided biopsy, surgery, radiotherapy plan, evaluation of fracture stability	Assessment of EMD	The gold standard in depicting diffuse bone marrow involvement can differentiate between pathological bone lesions and osteoporotic ones, corp compression
Prognostic relevant	Not clear	Prognostic significance of focal lesions number and SUV	Prognostic significance of focal lesions and diffuse pattern
Response/MRD evaluation	Not useful	Recommended	Possible alternative to PET-CT; data in progress

DWI, Diffusion-Weighted Imaging; EMD, Extramedullary Disease; FDG, 18F-Fluorodeoxyglucose; FL, Focal Lesion; IV, Intravenous; Min, Minutes; MRD, Minimal Residual Disease; mSv, Millisievert; RT, Radiotherapy; SUV, Standardized Uptake Value; WBLD CT, Whole-Body Low-Dose Computed Tomography.

#### Positron emission tomography (PET-CT)

Studies have shown that 18-fluorodeoxygenase (18F-FDG) PET-CT scans are a highly effective tool for identifying the localization of EMD lesions and assessing metabolic response to therapy [46]. Although MRI remains the gold standard for detecting plasma cell invasions in soft tissues, PET-CT provides complementary information, particularly in assessing bone marrow involvement where MRI may be limited or contraindicated [46]. This type of tomography has a higher sensitivity in detecting the localization of clonal plasmacytes, especially extramedullary ones. It exhibits higher performance in the initial staging of plasmacytomas, which correlates with MRI staging [47].

#### Magnetic resonance imaging (MRI)

MRI is a very important technique used for detecting lesions in soft tissue caused by extramedullary plasmacytoma. The most sensitive imaging technique for evaluating active multiple myeloma remains a whole-body MRI, as it provides more information about the characteristics of extramedullary plasmacytoma [48].

With its superior soft tissue contrast and multiplanar imaging capabilities, MRI is particularly valuable for evaluating non-osseous plasmacytomas, detecting spinal cord compression, and identifying lesions involving visceral organs or other soft tissues [49]. It is also used post-therapy to assess tumor reduction [9].

#### Computed tomography (CT)

CT helps assess bone lesions and is frequently used to evaluate MM cases. A better option is whole-body low-dose CT (WBLD-CT), which allows for the evaluation of the risk of pathological fractures and the presence of extramedullary lesions [50].

For monitoring patients and assessing suspected relapses, imaging is necessary to evaluate tumor growth, bone involvement, or organ damage. Currently, the first-choice imaging modality at diagnosis is WBLD-CT, while MRI or PET-CT is preferred in cases of relapse [51]. PET-CT has also been shown to provide valuable insights into clinical outcomes [52,53].

## MANAGEMENT AND TREATMENT OF EXTRAMEDULLARY DISEASE

### Radiotherapy (RT)

Radiotherapy is a treatment of choice and is frequently used in extramedullary disease. It achieves local control rates of approximately 80% in both solitary bone plasmacytomas and extramedullary plasmacytomas due to the radiosensitive nature of these lesions [35,54].

RT is typically delivered using linear accelerators that generate megavoltage beams. Patients usually receive a total dose ranging from 30 to 70 Gy, with the most common dose being 50 Gy. Larger tumors may require higher doses, generally around 50 Gy in 25 fractions, with each session delivering approximately 2 Gy [54,55].

### Surgical management

Surgical excision—partial or complete—can be an option in cases of extramedullary or solitary bone plasmacytomas. Surgery is often performed when patients present with a newly discovered tumor mass requiring histological evaluation. Studies suggest that combining surgery with radiotherapy leads to better outcomes [56]. However, surgery is typically reserved for feasible anatomical locations, as some areas, such as the head and neck, may be more radiosensitive, and surgical excision could result in mutilation or functional impairment [28]. Other indications for surgery are vertebral fracture, spinal cord compression, and vertebral instability [57]. In many cases, radiotherapy is administered after surgery to improve local control. Data suggest that omitting postoperative radiation therapy (RT) may increase the risk of recurrence [9].

### Systemic therapy – chemotherapy

In solitary plasmacytoma, no disease control or prevention of complications was shown when chemotherapy was used [35]. The only adjuvant chemotherapy that can be considered is for tumors greater than 5 cm or those unresponsive to radiotherapy [54]. Otherwise, chemotherapy has not been shown to reduce the incidence of progression to multiple myeloma but rather only to delay the time to progression [9]. Moreover, studies have shown that patients who received chemotherapy at the plasmacytoma stage had no survival benefit after progression to MM compared to those who did not receive early chemotherapy [58]. Exposure to early chemotherapy, when it is not necessary, exposes the patient to extra risks, including but not limited to the emergence of resistant subclones that limit the following therapeutic options or secondary neoplasia [58].

Evidence regarding the optimal treatment strategy for extramedullary disease remains limited due to the small number of dedicated studies [59]. However, patients with EMD who were not yet diagnosed with MM and who received novel agents, such as thalidomide, lenalidomide, or bortezomib, showed higher complete response rates compared to those who received standard therapies [60]. Some reviews propose treatment frameworks depending on transplant eligibility. For transplant-ineligible patients, combinations like daratumumab with VMP or RVD are recommended. Transplant-eligible patients may benefit from more intensive induction regimens such as VTD, VRD, or PACE, followed by stem cell transplantation (SCT) [59].

For relapsed patients, lymphoma-like regimens are suggested: PACE, DCEP, Dexa-BEAM, or HyperCVAD combined with autologous stem cell transplantation (ASCT)[61]. However, these regimens typically achieve only short-lived responses, with a median duration of less than 4 months. Emerging therapies, including pomalidomide, carfilzomib, selinexor, isatuximab, and novel agents such as chimeric antigen receptor T cells (CAR-T), bispecific T-cell engagers (BiTEs), and melflufen offer promising avenues for refractory cases [62]. Carfilzomib has shown superior efficacy in patients with bone-associated EMD compared to those with non-bone-related extramedullary involvement [63].

For central nervous system (CNS) involvement in EMD, which is very rare, radiotherapy and intrathecal chemotherapy are commonly used. Additionally, systemic therapies with immunomodulatory drugs (IMiDs) that can cross the blood-brain barrier (BBB) have proven to be a useful tool [64]; however, more data are needed to determine the optimal strategy [65]. Teclistamab, a bispecific antibody that targets CD3 receptors on T-cells and B-cell maturation antigen (BCMA) on myeloma cells, has shown low efficacy in patients with EMD [66,67]. However, when teclistamab is combined with talquetamab (a bispecific antibody targeting the G-protein coupled receptor GPRC5D on myeloma cells), outcomes appear to improve—particularly in EMD patients—with one study reporting an overall response rate (ORR) of 83% for this combination [68].

Previous data from prospective trials agreed that patients with extramedullary disease should be treated with aggressive therapies as a high-risk disease [42]. If patients are transplant-eligible, they recommend triplet induction therapy, followed by ASCT, and then triplet consolidation therapy, followed by maintenance treatment with lenalidomide or, instead of ASCT, a tandem ASCT was preferred by others [69]. For transplant-ineligible patients, VMP or continuous len-dex (lenalidomide and dexamethasone) are considered suitable and effective first-line treatments [70]. However, studies indicate that outcomes between standard-risk and high-risk patients are not significantly different [42]. It is important to note that EMD is rare, and when it presents as a solitary tumor, it is typically managed with systemic therapy. Consequently, most available data come from studies involving patients with multiple myeloma who later developed EMD [71]. EMD is linked with a poorer prognosis compared with MM without EMD; achieving a high response rate is essential but remains very challenging. Novel agents developed have shown greater efficacy in EMD compared with conventional chemotherapy. Lastly, response rates—defined by complete response (CR), very good partial response (VGPR), partial response (PR), and ORR—may vary between studies, which complicates their clinical interpretation and comparability [72]. An overview of systemic treatment options is presented in Table 4 [42,68,71,73-77].

**Table 4:** Systemic therapies response rate in extramedullary plasmacytoma or RRMM with EMD

Therapy/regimen	Patient population	Response rate	Key findings	Reference
Bortezomib-based therapies	RRMM with EMD	Often PR to near CR in small series	Bortezomib - more promising for EMD than single thalidomide	[71]
Bortezomib, dexamethasone, doxorubicin liposomal (BDD) followed by thalidomide/dexamethasone +/- bortezomib (BTD/TD)	RRMM with EMD (*n* = 14)	OS -86% (12/14) at 1 year	Efficacy of bortezomib regimens in improving poor prognosis of EMP	[73]
Pomalidomide-based regimens	Extramedullary MM (*n* = 6) - prior therapies used	Extramedullary ORR - 83% (5/6), CR - 50% (3/6), PR - 33% (2/6)	Promising activity in RR EMD	[74]
Isatuximab+ pomalidomide+ dexamethasone (Isa-Pd)	RRMM with EMD (14 pt in Isa-Pd vs. 10 in Pd)	ORR - 50% (7/14) in Isa-Pd vs 10% (1/10) in Pd	Efficacy in IsaPd in RRMM with EMD	[75]
Daratumumab (single)	RRMM with EMD ( 32% of 41 pt had EMD)	ORR - 24.4%	Limited efficacy on daratumumab for EMD - decreased CD38 expression	[75]
Bispecific antibodies (pooled analysis)	RRMM with EMD	ORR>48% (5 studies, 134 patients), pooled ORR across 14 studies (172 patients) -77% (Cl 68-87%)	Generally effective, with CAR-T showing superior OS	[42]
Talquetamab (bispecific antibody)	RRMM with EMD	ORR - 45% (Cl 17-77%)	Highest ORR among single bispecific antibodies for EMD	[76]
Teclistamab + talquetamab	RRMM with EMD	ORR - 71% (Cl 51-87%)	The only combination study to report ORR in EMD RRMM, high response	[76]
BCMA-CAR-T-cell therapy	RR Extramedullary plasmacytoma (*n* = 24)	Best ORR - 70,8%, CRR - 25%	Efficacy in RR EMP, long-term-survival suboptimal consolidation therapy may improve outcomes	[68]
CV-MED regimen (chidamine, bortezomib, mitoxantrone, etoposide, dexamethasone)	Case report - patient with EMP at progression	Remission of extramedullary lesion	Promising treatment strategy for EMD	[77]

CR Complete Response; CRR, Complete Response Rate; EMD, Extramedullary Disease; EMP, Extramedullary Plasmacytoma; ORR, Overall Response Rate; OS, Overall Survival; PR, Partial Response; RRMM, Relapsed/Refractory Multiple Myeloma.

## FOLLOW-UP

In patients with extramedullary involvement, regular follow-up is crucial to assess treatment response and detect early relapses. An initial reassessment of EMD using PET-CT or MRI should be performed 3 months after starting treatment. The physician should determine further imaging evaluations based on the patient’s progress. Using the same imaging modality as in the baseline assessment ensures consistency in monitoring [62]. A complete response is defined by the absence of any detectable extramedullary disease on imaging [52].

Minimal residual disease (MRD) assessment can be performed in addition to PET-CT to demonstrate double negativity and define a complete response [78]. The IMAJEM study (part of the IFM/DFCI 2009 trial) highlighted the value of MRI and FDG-PET/CT for baseline and post-treatment assessment in symptomatic multiple myeloma. Previous research has shown that MRD assessment serves as a strong biomarker for evaluating treatment efficacy and guiding future therapeutic decisions [79]. In high-risk newly diagnosed myeloma (NDMM) and relapsed/refractory MM, deeper responses, including MRD negativity, have been significantly correlated with improved progression-free survival (PFS) and overall survival (OS) [80]. When MRD is positive, even in patients with a complete response, it correlates with a shorter time to progression and vice-versa (a deep MRD negativity response is linked with better survival) [81].

MRD should be assessed at several key time points: at baseline, approximately day +100 following ASCT, and every 6 months thereafter if MRD negativity has been achieved [82]. MRD assessment is valuable in MM patients regardless of their baseline risk status due to its consistent correlation with PFS and OS [83]. Also, MRD has been used in trials to evaluate the new combinations of therapies as a marker of effectiveness in order to adapt treatment strategies [84]. The methods for MRD assessment are presented below.

### Next-generation sequencing

Next-generation sequencing (NGS) has a sensitivity of around 10^-5^ and can quickly assess multiple reads of DNA fragments, allowing it to distinguish clonal plasma cells from normal polyclonal B-cell populations. This capability provides detailed insight into disease heterogeneity and residual tumor burden [84].

### Flow cytometry-based methods

#### Multiparametric flow cytometry (MFC)

MFC is widely used to differentiate between normal and clonal plasma cells. It enables the quantification of malignant cells, the assessment of antigen expression levels (both surface and intracellular), and the characterization of various hematopoietic lineages. With a sensitivity range of 10^-4^ to 10^-3^, MFC remains an essential MRD detection tool. However, its performance may be limited by operator dependency and inter-laboratory variability [80].

#### Next-generation flow cytometry (NGF)

Next-generation flow cytometry (NGF) utilizes a standardized panel of 10 markers, including CD38, CD138, CD45, CD19, CD27, CD56, CD81, CD117, cytoplasmic Igκ, and cytoplasmic Igλ, which are analyzed across two tubes [85]. These markers enable precise discrimination between normal and clonal plasma cells. NGF is currently endorsed by the International Myeloma Working Group as the reference method for immunophenotypic assessment of minimal residual disease in multiple myeloma patients [85]. The cut-off for MRD negativity is 10^-5^, though recent trials have explored increasing the sensitivity threshold to 10^-6^ [86], which has shown improved predictive value for progression-free survival (PFS) [82]. The IMWG currently recommends NGF as the preferred method for MRD detection post-ASCT and in clinical trials, whereas MFC remains an accessible alternative in resource-limited settings [85,87].

#### Allele-specific oligonucleotide-PCR (ASO-PCR)

Allele-specific oligonucleotide-PCR (ASO-PCR) is useful because it is not affected by cell viability, unlike flow cytometry-based MRD techniques. It targets immunoglobulin heavy chain (IgH) gene rearrangements and known mutations. ASO-PCR achieves high sensitivity, typically reaching 10^-5^ and, in some settings, up to 10^-6^. Although ASO-PCR can achieve high sensitivity, reaching 10^-5^ and up to 10^-6^, it is not yet standardized in MM [81].

#### Droplet digital PCR

Droplet digital PCR is a more advanced molecular technique capable of detecting IgH rearrangements and disease-related mutations post-treatment. It offers greater precision than conventional quantitative PCR and does not require a standard calibration curve [88]. The criteria for a complete response in MM and EMD are presented in Table 5.

**Table 5:** IMWG MRD Criteria [89]

Requires a complete response (negative immunofixation on the serum and urine, disappearance of any soft tissue plasmacytomas, and <5% Plasma Cells in BM aspirates)	
Sustained MRD-negative	MRD-negativity in the marrow (NGF or NGS, or both) and by imaging, confirmed minimum of 1 year apart. Subsequent evaluations can be used to further specify the duration of negativity (e.g., MRD-negative at 5 years)
Flow MRD-negative	Absence of phenotypically aberrant clonal plasma cells by NGF on BM aspirates using the EuroFlow standard operation procedure for MRD detection in multiple myeloma (or validated equivalent method) with a minimum sensitivity of 1 in 10^5^ nucleated cells or higher
Sequencing MRD-negative	Absence of clonal plasma cells by NGS on BM aspirate in which the presence of a clone is defined as less than 2 identical sequencing reads obtained after DNA sequencing of BM aspirates using the LymphoSIGHT platform (or validated equivalent method) with a minimum sensitivity of 1 in 10^5^ nucleated cells or higher
Imaging plus MRD-negative	MRD-negativity as defined by NGF or NGS plus disappearance of every area of increased tracer uptake found at baseline or a preceding PET/CT or decrease to less mediastinal blood pool SUV or decrease to less than that of surrounding normal tissue

BM, bone marrow; DNA, deoxyribonucleic acid; IMWG, International Myeloma Working Group; MRD, minimal residual disease; NGF, next-generation flow; NGS, Next-Generation Sequencing; PET/CT, Positron Emission Tomography-Computed Tomography; SUV, Standardized Uptake Value.

## GENERAL CONSIDERATIONS AND CONCLUSION

Follow-up is crucial for patients with extramedullary disease or extramedullary plasmacytoma, as early detection and prompt management are essential for optimizing treatment and improving outcomes. It is important to recognize that when multiple myeloma relapses with EMD, it often becomes more resistant to therapy, making management significantly more challenging. Most cases require lifelong monitoring to prevent disease progression. Routine evaluations should include laboratory testing, such as M-protein levels, complete blood count, and biochemical panels every 6 weeks during the first 6 months and then at intervals determined by the patient’s clinical course. When a patient reports new or worsening bone pain, further investigations, such as imaging or additional diagnostic tests, should be performed to rule out relapse or progression to multiple myeloma. Patients with EMD require a multidisciplinary approach to ensure comprehensive evaluation and care. In addition to the core team, comprising a hematologist, radiation oncologist, and surgeon, other specialists may be needed based on the patient’s symptoms. These may include a neurologist, neurosurgeon, gastroenterologist, or ENT (ear, nose, and throat) specialist. It is also not uncommon for patients to initially present to an internal medicine specialist, which reinforces the importance of broad clinical awareness. The primary concern in patients with EMP is often not an immediate relapse but the risk of progression to multiple myeloma. This risk is influenced by factors such as age and the nature of the disease at the time of presentation. Multiple myeloma with extramedullary involvement can present with atypical or unusual symptoms, which may complicate diagnosis and delay treatment. Therefore, patients with EMD or EMP require thorough and attentive evaluation by all specialists involved in their care. Comprehensive diagnostic workups can provide critical information and help avoid delays in identifying disease progression or relapse. A coordinated, multidisciplinary approach is crucial for achieving optimal outcomes in both the evaluation and ongoing management of patients with extramedullary disease.

## Data Availability

Further data is available from the corresponding author upon reasonable request.
